# Barriers and enablers to non‐remunerated plasma donation: A meta‐synthesis of the qualitative literature using the theoretical domains framework

**DOI:** 10.1111/vox.70312

**Published:** 2026-07-02

**Authors:** Cole Etherington, Ashutosh Upreti, Amira Brehaut, Megan Bennett, Maximillian Labrecque, Kyle Rubini, Emily Gibson, Manavi T. Maharshi, Amelia Palumbo, Samantha Meyer, Terrie Foster, Joyce Dogba, Risa Shorr, Vivian Welch, Justin Presseau, Elisabeth Vesnaver

**Affiliations:** ^1^ Methodological and Implementation Research Program, Ottawa Hospital Research Institute Ottawa Ontario Canada; ^2^ Lived Experience Partner Calgary Alberta Canada; ^3^ Lived Experience Partner London Ontario Canada; ^4^ School of Psychology, University of Ottawa Ottawa Ontario Canada; ^5^ School of Public Health Sciences, University of Waterloo Waterloo Ontario Canada; ^6^ Canadian Blood Services Ottawa Ontario Canada; ^7^ VITAM – Sustainable Health Research Centre Quebec City Quebec Canada; ^8^ Learning Services, The Ottawa Hospital Ottawa Ontario Canada; ^9^ School of Epidemiology and Public Health, University of Ottawa Ottawa Ontario Canada; ^10^ Bruyère Health Research Institute Ottawa Ontario Canada; ^11^ BORN Ontario, Children's Hospital of Eastern Ontario Ottawa Ontario Canada

**Keywords:** barriers and enablers, donor recruitment and retention, plasma donation, plasmapheresis, qualitative meta‐synthesis, theoretical domains framework

## Abstract

**Background and Objectives:**

Plasma is an essential component of therapies used to treat immune deficiencies, coagulation disorders and other serious conditions. Despite increasing global demand, many countries, including Canada, remain dependent on imported plasma products. Understanding the psychological, social and structural factors influencing plasma donation is critical to designing strategies that strengthen domestic supply. This meta‐synthesis aimed to identify barriers and enablers to plasma donation using the theoretical domains framework (TDF).

**Materials and Methods:**

A systematic search of Medline, Embase, Scopus, PsycINFO and CINAHL (up to August 2023) identified qualitative studies describing experiences and perceptions of plasma donation. Using directed content analysis, qualitative data were coded to TDF domains, and belief statements summarizing barriers and enablers were generated and synthesized narratively.

**Results:**

Twenty studies (*n* = 1310 participants) conducted in non‐remunerated contexts met inclusion criteria. A total of 131 belief statements were mapped to 13 of 14 TDF domains. The largest number occurred in *Environmental Context and Resources* (e.g., accessibility, scheduling, staff support), followed by *Knowledge*, *Social/Professional Role and Identity*, *Social Influences*, and *Emotion*. Barriers centred on logistical constraints, limited understanding and negative emotional responses, whereas enablers reflected trust in institutions, supportive staff, identity as a donor and feelings of pride and altruism.

**Conclusion:**

Plasma donation behaviour is shaped by interacting informational, emotional, social and structural determinants. Addressing practical barriers while leveraging enablers such as donor identity, social support and transparent communication may enhance recruitment and retention. Our findings can inform the design of theory‐based, donor‐centred interventions to strengthen national plasma collection systems.

**Highlights**

- Barriers and enablers to plasma donation were identified across 13 of 14 theoretical domains framework (TDF) domains.
- Key determinants were environmental context and resources, especially accessibility, scheduling and staff support, followed by knowledge, donor identity, social influences and emotion.
- Efforts to close informational gaps, reduce structural barriers and strengthen inclusive donor engagement may support long‐term national plasma sufficiency.

## INTRODUCTION

Plasma‐derived medicinal products (PDMPs), such as immunoglobulins, are essential therapies for the treatment of immune deficiencies, bleeding disorders and various neurological and autoimmune conditions [1, 2, 3]. Plasma used to manufacture PDMPs can be obtained either as recovered plasma separated from whole‐blood donations or as source plasma collected directly through plasmapheresis [4]. In both cases, the plasma undergoes industrial fractionation to isolate specific proteins for therapeutic use. Internationally, a substantial proportion of plasma for fractionation is collected as source plasma, and growing demand for PDMPs has intensified pressure on plasma supply systems [5]. For countries that rely heavily on imported PDMPs, this creates vulnerability to international market disruptions and has strengthened interest in expanding domestic source plasma collection [6, 7]. In response, national blood operators in several settings have expanded plasma‐only collection centres, launched public information campaigns and sought to encourage whole‐blood donors to convert to source plasma donation [8, 9].

Although recovered plasma also contributes to PDMP manufacture, expanding source plasma collection presents distinct operational and behavioural challenges [10, 11, 12, 13, 14, 15, 16]. In many blood systems, donor recruitment, retention and service models were developed primarily around whole‐blood donation rather than industrial‐scale source plasma collection. As a result, efforts to increase source plasma supply are unlikely to be fully supported by strategies derived from whole‐blood donation alone and require a clearer understanding of the factors influencing source plasma donation specifically [10, 11, 12, 13, 14, 15, 16].

Compared with whole‐blood donation, source plasma donation via plasmapheresis typically requires a longer appointment and involves different procedural features (e.g., blood is circulated through an apheresis device, plasma is collected and cellular components are returned to the donor). These features can create distinct experiential and logistical considerations, such as time burden, comfort during the return cycle and reactions related to anticoagulant use [16, 17, 18]. At the same time, source plasma donation may offer different advantages, including greater donation frequency. These differences suggest that plasma donation behaviour may be shaped by motivational, cognitive, emotional, social and contextual influences that are not fully captured by evidence from whole‐blood donation alone [16, 17, 18].

Quantitative evidence, including a recent meta‐analysis, has identified several key determinants of plasma donation intention and behaviour, including beliefs about capability, social influences and emotions [19]. Quantitative approaches are well suited to estimating associations between pre‐specified determinants and donation outcomes, but they are less able to explain how these determinants are experienced, interpreted and enacted within donors' everyday lives and broader social contexts. Qualitative studies can therefore complement quantitative evidence, providing insight into donors' lived experiences, perceptions and decision‐making processes, and are particularly well suited to identifying influences that may not yet be captured within existing theoretical or quantitative frameworks. Synthesizing qualitative evidence may therefore not only deepen understanding of known determinants but also identify novel relational, contextual and experiential factors shaping plasma donation behaviour. The present review aimed to identify and synthesize barriers and enablers to plasma donation reported in qualitative studies using the theoretical domains framework (TDF). TDF integrates constructs from 33 behaviour‐change theories into 14 domains encompassing cognitive, emotional, social and environmental influences on behaviour and has associated tools that link to theory‐informed intervention development [20, 21]. TDF was selected to guide this synthesis given that its breadth allows diverse qualitative findings to be organized systematically while facilitating future theory‐informed intervention development. This approach enables a structured interpretation of qualitative evidence while preserving attention to the contextual and experiential factors that shape plasma donation behaviour.

## MATERIALS AND METHODS

### Protocol and registration

The protocol for this review was registered [22] and published a priori [22]. The protocol originally included an intersectionality informed analysis; however, this component was removed after data extraction because reporting within the included studies was not sufficiently detailed to support analysis across intersecting social categories. All other steps were conducted as planned.

### Information sources and search strategy

An information specialist developed a comprehensive peer‐reviewed [23] search strategy in collaboration with the research team (File S1). Electronic searches were conducted in Medline, Embase, Scopus, PsycINFO and CINAHL from database inception to 23 August 2023. Reference lists of all included studies were screened to identify additional sources.

### Eligibility criteria

Studies were eligible for inclusion if they (1) collected qualitative data (e.g., interviews, focus groups) describing experiences, perceptions or beliefs about plasma donation; (2) included participants who were potential, current or former plasma donors (source or convalescent; paid or unpaid); and (3) were published in English or French in a peer‐reviewed journal. Although convalescent plasma differs in clinical purpose, the behavioural act of donation involves shared procedural, physical and logistical characteristics [24]. Studies focused exclusively on whole‐blood donation, as well as commentaries, conference abstracts and theses, were excluded.

### Study selection

Search results were imported into DistillerSR (Evidence Partners, Ottawa, Canada) for management and screening. Two reviewers independently screened all titles, abstracts and full texts using pre‐piloted forms. Disagreements were resolved through discussion or consultation with a third reviewer.

### Data extraction

Two reviewers independently extracted descriptive information (author, year, country, participant characteristics and study design) and all qualitative findings, including first‐order data (participant quotations) and second‐order data (thematic interpretations reported by original study authors). Second‐order data were treated strictly as the analytical interpretations provided by the primary study authors.

During synthesis, quotations and author‐assigned interpretations were each coded, but each data element was ultimately assigned to a single final domain to prevent double‐counting. No discrepancies were identified between the meaning conveyed in participants' quotations and how they were interpreted in the original studies. All extracted data were cross‐checked by a third reviewer for accuracy and completeness, and reviewer discussions ensured consistency in interpretation throughout the process.

### Data synthesis

Data were analysed using directed content analysis guided by TDF (File S2). Directed content analysis is a deductive qualitative approach in which existing theory or prior research guides the development of initial coding categories [25]. This approach was appropriate because TDF provided an a priori framework for organizing barriers and enablers across studies while allowing reviewers to interpret how qualitative data mapped onto theoretically defined behavioural domains. Two reviewers independently coded all qualitative data to one or more of the 14 TDF domains using a coding manual developed by the research team [19]. Coding discrepancies were discussed until consensus was achieved. During synthesis, each data element was ultimately assigned to the single final domain judged by reviewers to best reflect the primary behavioural mechanism represented in the original data. This was done to support parsimony, consistency, interpretability, and to avoid double‐counting or inflating domain frequencies. This coding decision aligns with TDF guidance recommending that text be coded to the domain that best reflects the key theme, with multiple domain coding used only when consensus on a single primary domain cannot be reached [21]. It is also consistent with prior TDF work that used primary‐domain coding [26, 27].

After coding, one reviewer generated belief statements summarizing barriers and enablers within each TDF domain. These belief statements captured the key ideas underlying participant quotations and author interpretations. A second reviewer independently reviewed all belief statements for accuracy and completeness, and disagreements were resolved through discussion. The finalized set of belief statements was then synthesized narratively to identify recurring behavioural determinants and describe how they mapped onto the TDF domains.

## RESULTS

### Study and participant characteristics

**FIGURE 1:**
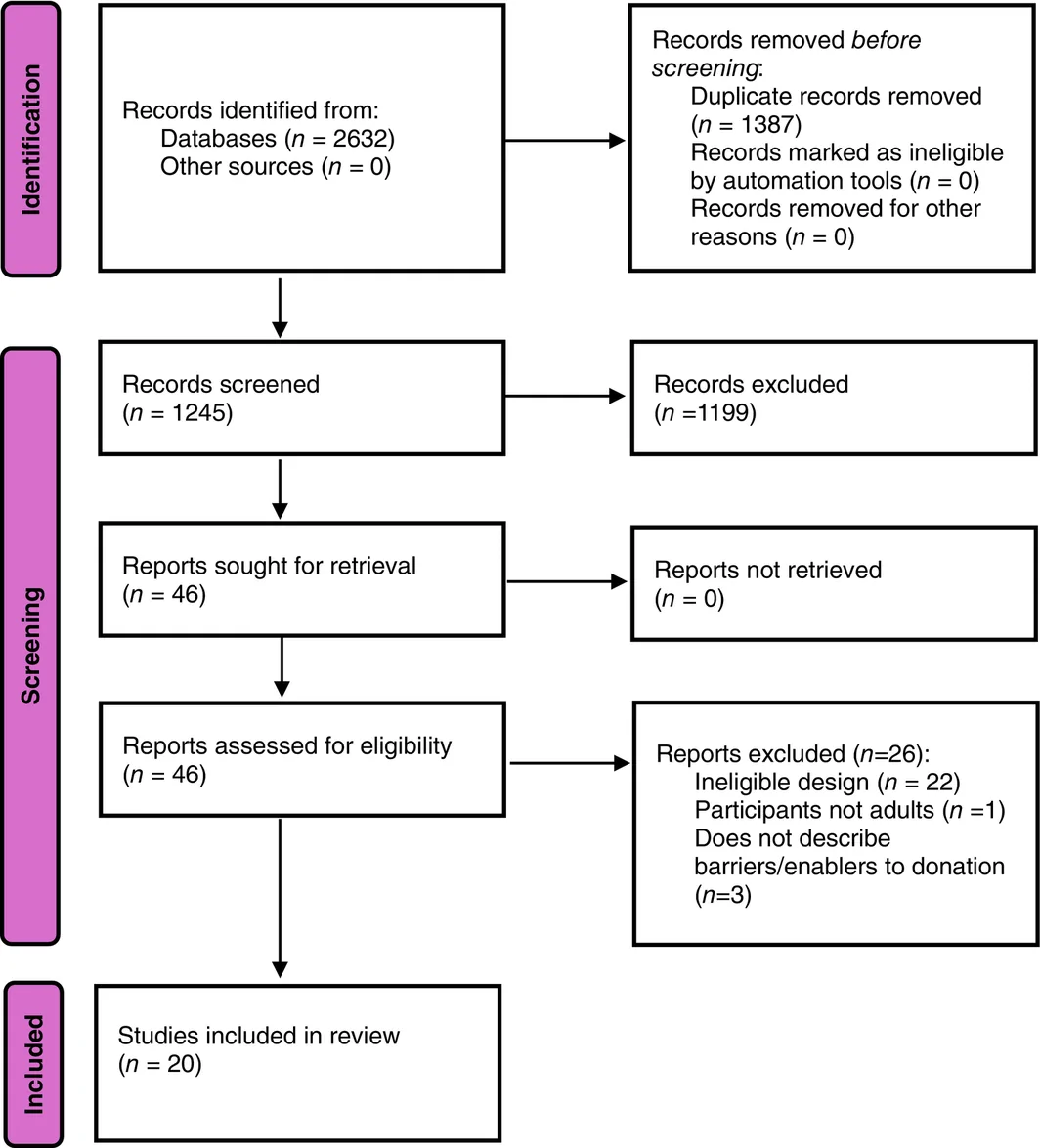
Preferred reporting items for systematic reviews and meta‐analyses (PRISMA) flow diagram.

Twenty qualitative studies (Figure 1) published between 2004 and 2023 were included, spanning Australia [14, 15, 17, 28, 29, 30, 31, 32], Canada [12, 33, 34, 35, 36, 37], France [38, 39], Guinea [40, 41], Poland [42] and Peru [43] (Table S1). The methods used by most studies involved focus groups or semi‐structured interviews, with several employing narrative or ethnographic approaches. Six studies drew on theoretical frameworks including the theory of planned behaviour [33, 38], grounded theory [14, 42], identity theory [29] and critical realist approaches [31].

**FIGURE 2:**
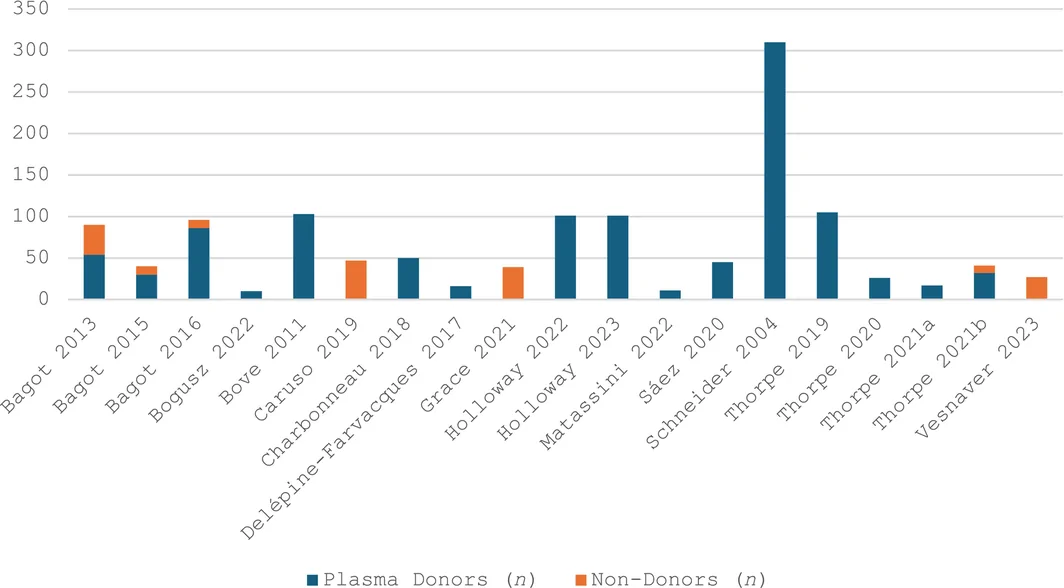
Representation of plasma donors versus non‐donors across included studies. Ronse et al. [40] included both donors and non‐donors but did not report frequencies of each. Active, first‐time or newly converted and lapsed (previously active) donors were included as plasma donors. Non‐donors were considered to be those who never donated, whether or not they were eligible or restricted from donating.

Across the 20 included studies, 15 included only plasma donors, 3 included only non‐donors and 2 included both donors and non‐donors (Figure 2). Among studies of donors, participant groups included whole‐blood donors invited to convert to plasma donors [15, 17, 28, 29], regular or high‐frequency plasma donors [14, 32], lapsed donors [31] and specialized programmes such as anti‐D and convalescent plasma donors [44]. Several studies focused on specific subgroups, including gay, bisexual, queer and other men who have sex with men [12, 33, 35] and Ebola survivors participating in convalescent plasma programmes [40, 41]. The combined sample size was 1310 participants, with a median of 44.5 (range 10–310). Participants were recruited primarily through national blood services or affiliated donor centres, such as Australian Red Cross Lifeblood, Canadian Blood Services, Héma‐Québec and Établissement français du sang, with a smaller number of studies situated in hospital or outbreak contexts [40, 41, 43]. No included study sampled commercially paid donors. The two Ebola studies from Guinea involved an emergency trial context where material support was described; however, this was not within a paid/commercial plasma donation model [40, 41].

Reporting of participant demographics was variable across studies, with many categories inconsistently described (File S3). Most samples included adults spanning young to older age, typically skewing towards middle‐aged participants. Age was reported in 17 studies, with participants' pooled mean ages generally in the late 30s to late 40s. Gender was reported in 12 studies; across pooled counts, approximately 546 participants identified as men and 355 as women. Five studies also included small numbers of trans, non‐binary, queer or genderfluid participants. Education was reported in seven studies and most often reflected post‐secondary or university‐level attainment. Ethnicity appeared in five studies, with the majority identifying as White or European and smaller proportions as Black, Indigenous, Latino, South Asian or other backgrounds. Sexual orientation was explicitly reported by the three studies focused on gay, bisexual and other men who have sex with men (gbMSM) donors [12, 33, 35]. Employment, income and marital or household status were occasionally reported but with limited consistency.

### TDF results

#### Overview of TDF domains

**FIGURE 3:**
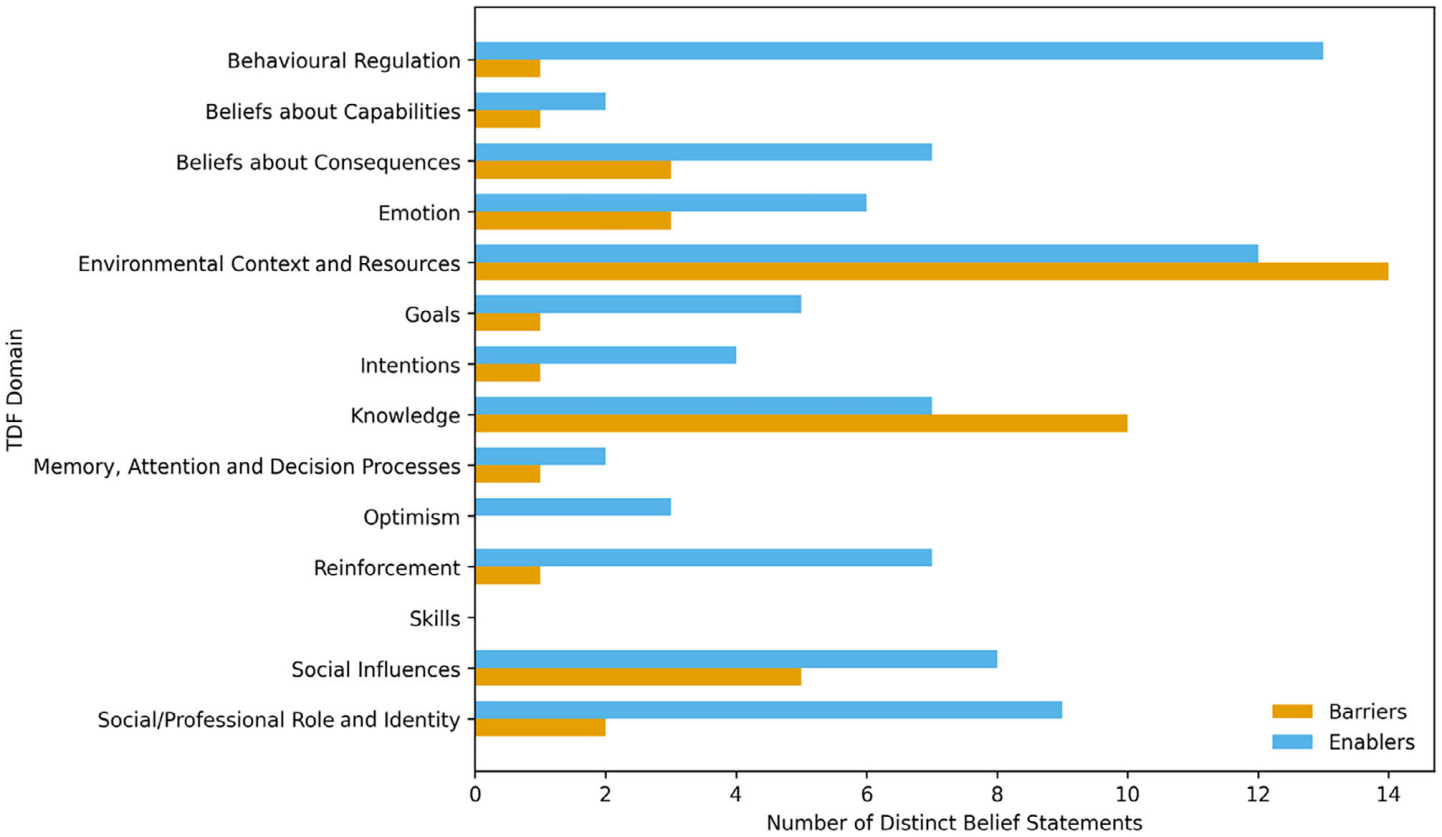
Barriers and enablers to plasma donation by theoretical domains framework (TDF) domain.

Across studies, a total of 131 belief statements were identified (Table S2) and mapped to 13 of the 14 domains of the TDF (Figure 3). No statements were coded to the *Skills* domain. For each domain, *n* refers to the number of distinct belief statements, and *f* represents the cumulative frequency with which those beliefs appeared across studies.

The largest number of statements were identified in *Environmental Context and Resources* (*n* = 26, *f* = 129), followed by *Knowledge* (*n* = 17, *f* = 87), *Beliefs about Consequences* (*n* = 10, *f* = 72), *Social/Professional Role and Identity* (*n* = 11, *f* = 59), *Social Influences* (*n* = 13, *f* = 60) and *Emotion* (*n* = 10, *f* = 61). Moderate representation was observed in *Behavioural Regulation* (*n* = 14, *f* = 35) and *Reinforcement* (*n* = 8, *f* = 29), while smaller domains included *Intentions* (*n* = 6, *f* = 12), *Goals* (*n* = 6, *f* = 11), *Optimism* (*n* = 3, *f* = 11), *Beliefs about Capabilities* (*n* = 3, *f* = 8) and *Memory, Attention and Decision Processes* (*n* = 3, *f* = 6). Overall, barriers were largely concentrated in the domains of *Environmental Context and Resources*, *Knowledge* and *Emotion*, while enablers were more common in *Social/Professional Role and Identity*, *Reinforcement* and *Behavioural Regulation* (Figure 2).

#### Environmental context and resources (*n* = 26, *f* = 129)

Practical circumstances and institutional settings were frequently described by participants as affecting their plasma donation decisions. Barriers (*n* = 14, *f* = 86) included difficulty scheduling appointments (‘I find it difficult to schedule appointments because of work or family’; *f* = 12), limited accessibility or long travel times (*f* = 8, 5) and the perceived time burden of plasma donation (‘Plasma donation takes too long compared to whole blood’; *f* = 10). Participants also noted frustration with administrative processes, restrictive eligibility and discouraging staff interactions. Concerns about trust, commercialization and policy‐related stigma were also described. Enablers (*n* = 11, *f* = 43) included convenient scheduling, accessible and well‐organized centres and professional, supportive staff (‘Staff make me feel welcome and comfortable’; *f* = 10). Participants described how inclusive eligibility rules and trust in voluntary, non‐remunerated systems reinforced confidence in donating (*f* = 3–5).

#### Knowledge (*n* = 17, *f* = 87)

Knowledge was described as shaping engagement in plasma donation through both uncertainty and understanding. Barriers (*n* = 10, *f* = 63) were reflected in limited understanding of the plasmapheresis process, including uncertainty about procedural steps such as blood return and use of needles (‘I don't know how the plasmapheresis process works’; *f* = 12), and limited awareness of the purpose or need for plasma (‘I don't know what plasma is or why it is needed’; *f* = 14). Participants also described a lack of knowledge regarding eligibility and donation frequency (‘I don't know the rules about who can donate plasma, how often, or whether I can switch donation types’; *f* = 10), possible side effects (‘I don't know how plasma donation will affect my body or my ability to do physical activity afterwards’; *f* = 2) and how plasma is used or distributed (‘I want more transparency about how plasma is used, where it goes, how it is sold, and what happens with profits’; *f* = 5). Some accounts also noted limited knowledge of ethical and policy issues, such as HIV screening or for‐profit models.

Enablers (*n* = 7, *f* = 24) described how understanding procedures and uses of plasma supported donation. Participants reported that familiarity with donation steps helped them to feel at ease (‘When I understand the procedure, I feel less anxious and more confident about donating’; *f* = 5), learning about the uses of plasma encouraged participation (‘When I learn about the uses of plasma [e.g., medicines, treatments], I am more willing to donate’; *f* = 6), and transparency about plasma use provided reassurance (‘When I understand where plasma goes and how it is used, I feel more confident about donating’; *f* = 4).

#### Beliefs about consequences (*n* = 10, *f* = 72)

Participants described a range of beliefs about the outcomes of plasma donation. Barriers (*n* = 3, *f* = 33) included concern about contamination or infection (‘I worry about contamination, infection, or permanent injury from plasma donation’; *f* = 11), physical side effects such as dizziness or fatigue (‘I experience or fear physical side effects …’; *f* = 15) and negative health impacts (‘I believe plasma donation may weaken my body or immunity’; *f* = 7).

Enablers (*n* = 7, *f* = 39) reflected minimal side effects or positive experiences (‘I felt fine afterwards and would do it again’; *f* = 6) and beliefs about the positive impacts of donation (‘Plasma donation helps others and saves lives’; *f* = 9). Participants described donation as contributing to the health of others and to collective medical progress.

#### Social/professional role and identity (*n* = 11, *f* = 59)

Across studies, plasma donation emerged as part of how individuals defined themselves and related to others. Enablers (*n* = 8, *f* = 39) reflected a perception of donation as part of who one is or what one contributes to society (‘I see myself as a donor … and this identity motivates me to continue donating’; *f* = 9). Participants described pride and recognition (‘I feel recognized and valued for being a donor’; *f* = 7) and sometimes connected donation to family or community traditions (‘People close to me are donors or recipients, and this influences how I see my role’). For some, donation aligned with professional or civic responsibility. Barriers primarily involved stigma or lack of recognition. Participants from marginalized groups, particularly gbMSM donors, described exclusion from donation as inconsistent with their self‐image and community values (‘Being in a marginalized group … means my role as a donor is not fully recognized’; *f* = 8). Mixed influences included demographics (e.g., donation being linked to age, gender or social background; *f* = 7) and trust/authority, which motivated some donors but discouraged others who felt their community's contributions were undervalued (*f* = 4).

#### Social influences (*n* = 13, *f* = 60)

Social influences were described as both facilitating and discouraging plasma donation. Enablers (*n* = 8, *f* = 38) included encouragement and recognition from family, friends and staff (‘My family supports me donating’; *f* = 3; ‘Staff encouragement helps me keep donating’; *f* = 7). Community norms and a sense of shared purpose also motivated donation (‘Donation is something people in my community do’; *f* = 4–5). Barriers (*n* = 5, *f* = 22) related to stigma and lack of recognition, particularly among gbMSM donors (‘I feel people disapprove because of my sexual orientation’; *f* = 6). Participants described feeling discouraged when staff or peers showed disinterest or criticism (*f* = 4–5).

#### Emotion (*n* = 10, *f* = 61)

There was a range of emotional reactions associated with plasma donation. Barriers (*n* = 3, *f* = 29) included fear of needles or contamination (‘I feel anxious or panicked about donating’; *f* = 12), discomfort during the procedure (‘I feel distressed by the physical sensations’; *f* = 10) and uncertainty linked to limited information (*f* = 7). Enablers (*n* = 7, *f* = 32) included relief after successful donation, feelings of pride and gratitude (‘I feel proud knowing my plasma helps others’; *f* = 5–7) and reassurance from staff (‘Staff reassurance makes me feel calm’; *f* = 5).

#### Behavioural regulation (*n* = 14, *f* = 35)

Behavioural regulation was discussed in relation to how participants maintained regular plasma donation. Enablers (*n* = 13, *f* = 33) included developing habits and routines (‘I've developed a habit of donating plasma regularly’; *f* = 7), scheduling donations at regular intervals (*f* = 3) and using reminders or mobile apps (‘I am more likely to donate when I receive clear, timely reminders’; *f* = 6). Participants also mentioned that structured systems and supportive processes helped sustain their engagement. The only barrier (*n* = 1, *f* = 2) referred to disruptions of established routines due to life changes.

#### Reinforcement (*n* = 8, *f* = 29)

Reinforcement of plasma donation appeared through feedback and recognition. Enablers (*n* = 7, *f* = 27) included appreciation from staff (‘I feel encouraged when staff thank me or acknowledge my donations’; *f* = 6), small rewards or tokens (‘I appreciate the recognition of milestones’; *f* = 3) and awareness of the impact of their contributions. Expressions of gratitude or achievement reinforced continued participation. The only barrier (*n* = 1, *f* = 2) reflected concern that tangible rewards could detract from patient care or the altruistic nature of donation.

#### Intentions (*n* = 6, *f* = 12)

Intentions were described through participants' statements about their plans and readiness to donate plasma. Enablers (*n* = 4, *f* = 9) included a belief that donation was a good thing to do (‘I think donating is a good thing and I want to keep doing it’; *f* = 3), willingness to donate when invited (‘I donate when I'm asked to’; *f* = 2) and intention to return after positive experiences. Barriers (*n* = 1, *f* = 2) related to time and competing commitments (‘I'm often too busy or lack spare time, so donation isn't a priority’; *f* = 2). One neutral account reflected spontaneous participation (‘I don't feel I need a reason to donate, I just do it when it comes up’; *f* = 1).

#### Goals (*n* = 6, *f* = 11)

For many donors, plasma donation represented a personal goal to uphold or achieve over time. Enablers (*n* = 5, *f* = 10) included setting targets (‘I set personal goals for the number of donations I want to achieve’; *f* = 3), achieving milestones (‘Reaching my target feels rewarding’; *f* = 2) and linking goals to helping others (‘I want to donate to help those who need it’; *f* = 2). The only barrier (*n* = 1, *f* = 1) reflected disappointment when unable to meet self‐set goals (‘I feel disappointed when I can't meet the donation goals I set’; *f* = 1).

#### Memory, attention and decision processes (*n* = 3, *f* = 6)

Participants described how reminders and decision cues affected their plasma donation behaviour. Barriers (*n* = 2, *f* = 4) included forgetting to donate (‘I don't usually remember to donate unless I'm reminded’; *f* = 2) and being too busy to keep donation top of mind (*f* = 2). Enablers (*n* = 2, *f* = 4) included regular reminders and decision support (‘Regular reminders help keep donation salient’).

#### Beliefs about capabilities (*n* = 3, *f* = 8)

Beliefs about personal ability influenced how participants described their engagement with plasma donation. Enablers (*n* = 2, *f* = 6) reflected confidence gained through experience (‘Plasma donation is as easy as or easier than whole blood once you're used to it’; *f* = 5) and comfort with routine participation (‘I am inspired to return when the process feels easy’; *f* = 1).Barriers (*n* = 1, *f* = 2) involved physical limitations (‘I struggle to donate because my veins are small or difficult to find’; *f* = 2).

#### Optimism (*n* = 3, *f* = 11)

Optimism was described as an enabling influence on plasma donation. Participants expressed optimism when they perceived their contribution as especially valuable (‘I feel optimistic because my blood type or plasma is especially valuable to others’; *f* = 1) or when donation programmes were seen as symbols of progress and inclusion (‘I feel optimistic because new programmes represent progress and reduced stigma’; *f* = 5). Some participants expressed hope that plasma programmes could help reduce prejudice and improve access to donation (*f* = 5).

## DISCUSSION

This qualitative meta‐synthesis examined barriers and enablers to plasma donation using TDF. By organizing existing qualitative evidence within this framework, the review provides an integrated understanding of the psychological, social and contextual factors shaping plasma donation. Determinants were represented across nearly all TDF domains, highlighting the behavioural complexity of donation and the interplay between informational, emotional, social and structural influences.

The *Environmental Context and Resources* domain contained the largest number of belief statements, indicating that most barriers stemmed from logistical and institutional conditions such as accessibility, scheduling and staff capacity. This was followed by *Knowledge*, *Social/Professional Role and Identity*, *Social Influences* and *Emotion*, which together captured much of the variation in how donors understood and sustained participation. Barriers were concentrated in the environmental domain, whereas enablers were more common elsewhere, suggesting that while structural constraints often limited donation, psychological and social processes, including confidence, belonging and appreciation, can help sustain engagement. The absence of belief statements coded to the *Skills* domain likely reflects the nature of plasma donation from the donor perspective, as donors generally do not need procedural skills to donate plasma.

Across studies, donors emphasized the importance of clear information, supportive staff and trust in collection systems. Understanding procedures, feeling valued and perceiving transparency around how plasma is used encouraged continued participation, whereas uncertainty, time pressure and perceived exclusion discouraged it. These determinants are likely to remain relevant in the current context of increasing demand for PDMPs, renewed emphasis on domestic plasma sufficiency and rapid expansion of plasma collection efforts. As blood operators increasingly invest in dedicated programmes, public awareness and familiarity with plasma donation are likely to evolve, potentially shifting the relative importance of this determinant over time. Scaling plasma collection and achieving sufficiency depends on both recruitment of new donors and donors returning regularly. This makes identified factors such as accessibility, staff support, clear communication, emotional experience and donor identity central to contemporary plasma recruitment and retention efforts. These findings complement quantitative evidence identifying beliefs about capability, social influence and emotion as key correlates of intention [19]. The present synthesis helps in explaining how these constructs manifest in practice: intentions are enacted within donors' lived contexts, shaped by personal confidence, social support, emotional experience and the operational environment of donation systems. Importantly, most included studies (17/20) included current or former plasma donors, meaning that most of the determinants identified in this review were informed by lived experience of having donated. As such, many identified enablers and barriers could be interpreted as directly related to donation behaviour itself. At the same time, no included study sampled renumerated plasma donors, meaning the evidence reviewed may not generalize to paid plasma collection settings. This is an important gap to address, as remuneration may alter motivational structures, perceptions of altruism, trust in collection systems and the relative importance of financial, logistical and relational factors.

Recent qualitative work has underscored the relational dimensions of plasma donation. Seeley et al. [45] found that for new plasma donors in Canada, intentions to continue donating were shaped by experiences of ‘reciprocal care’—feeling cared for by attentive staff and, in turn, caring for others through donation. Staff interactions, feelings of well‐being and integration of donation into everyday life formed a broader web of relationships sustaining engagement. These findings reinforce our synthesis that emotional and social processes, including reassurance, belonging and perceived appreciation, operate as key enablers within TDF domains such as *Social Influences*, *Emotion* and *Environmental Context and Resources*.

Persistent gaps remain in how participant characteristics and study contexts are reported in plasma donation research. Demographic information was often limited, and when provided, categories were inconsistently defined. These omissions restrict examination of variation across subgroups and settings and limit the capacity of national blood operators to design responsive, inclusive strategies. Improved reporting standards, particularly consistent and transparent demographic data, would strengthen the interpretability and applicability of qualitative findings. Although our protocol initially included an intersectionality informed analysis, this component was not undertaken because most studies lacked sufficient data on intersecting social identities or contextual factors. This limitation reflects a broader issue in the evidence base and will be addressed in a forthcoming commentary examining how intersecting social identities shape experiences and behavioural determinants of plasma donation across our related systematic reviews [19].

The limited attention to identity‐related factors in the plasma donation literature is notable given broader blood donation research showing that donor identity, eligibility policies, stigma and institutional recognition can shape whether people feel able or invited to donate [46, 47, 48, 49]. Although plasma and whole‐blood donation differ procedurally, many identity‐relevant processes occur through shared institutional pathways, including donor screening, eligibility assessment, communication and interactions with staff. For groups historically excluded or marginalized by blood donation policies, donation may be experienced not only as a health behaviour but also as a site where belonging, trust, civic participation and recognition are affirmed or undermined. Future plasma donation research should therefore examine how social identity, structural exclusion and institutional trust shape willingness and ability to donate rather than treating donor characteristics only as demographic descriptors.

This review applied a theory‐based framework to systematically identify behavioural determinants of plasma donation across diverse qualitative designs. Its findings suggest that plasma donation is a complex behaviour shaped by interacting informational, emotional, relational, structural and institutional factors. Accordingly, TDF domain assignments should be interpreted as a structured analytic aid for identifying salient behavioural mechanisms, rather than as evidence that determinants are discrete or isolated. Some determinants could plausibly be relevant to more than one domain, particularly where donors described emotional responses shaped by information, staff interactions, or perceptions of risk. Frequency counts (*n* and *f*) were therefore used descriptively to indicate relative emphasis, not as quantitative measures of determinant strength. Findings also reflect the scope and quality of available studies, most of which originated in high‐income settings and drew on small, demographically narrow samples, with limited inclusion of first‐time donors or non‐donors.

Given this complexity, efforts to strengthen plasma donor recruitment and retention will likely require multi‐component interventions rather than single‐strategy approaches. Clear, transparent communication about plasmapheresis procedures, eligibility and the use of plasma may help reduce uncertainty and build trust, while supportive, well‐trained staff can create positive emotional experiences and reinforce donors' sense of contribution and belonging. Structural supports, such as flexible scheduling, accessible centres and efficient appointment systems, can lessen burdens related to time and convenience. Behavioural interventions should target relevant TDF domains, such as *Knowledge*, *Emotion* and *Environmental Context and Resources* while leveraging enablers identified within *Social Influences* and *Identity*. Implementation‐science frameworks like the Behaviour Change Wheel and COM‐B model can guide translation of these determinants into targeted, evidence‐based strategies. Future research should prioritize diversity and inclusion, employing mixed‐methods and participatory approaches that link behavioural theory with lived and living experience.

This qualitative synthesis advances understanding of the cognitive, social, emotional and contextual influences on plasma donation. Findings complement quantitative evidence by demonstrating how plasma donation determinants are experienced and enacted within donors' relationship, identities, institutional experiences and broader social contexts. By systematically organizing determinants within TDF, it provides a foundation for theory‐informed, donor‐centred interventions. Addressing informational gaps, structural barriers and inclusivity in donor engagement will be essential to achieving and sustaining national plasma sufficiency goals.

## CONFLICT OF INTEREST STATEMENT

The authors declare no conflicts of interest.

## Supporting information


**File S1.** Search strategies.

**File S2.** Definitions of theoretical domains framework domains.

**File S3.** Full demographic summary and age range by study.

**Table S1.** Characteristics of included studies.
**Table S2.** Belief statements on plasma donation by theoretical domains framework (TDF) domain.

## Data Availability

The data that support the findings of this study are available from the corresponding author upon reasonable request.
