# Supplementary material for: Barriers and enablers to non‐remunerated plasma donation: A meta‐synthesis of the qualitative literature using the theoretical domains framework
Source: Vox Sang. 2026 Jul 2;121(9):1290–300. doi: 10.1111/vox.70312 (PMC13563490; doi:10.1111/vox.70312)
Supplement: Supplementary file 1 [file VOX-121-1290-s001.docx]

**Supplementary File 4**

**Supplementary Table 1. Characteristics of included studies**

| **Author (Year)** | **Country** | **Study design/analytic approach** | **Sample size (n)** | **Study setting** | **Study population** |
| --- | --- | --- | --- | --- | --- |
| Bagot et al. (2013) | Australia | Focus groups (9) and individual interviews (6); thematic analysis | 90 | Australian Red Cross Blood Service (metropolitan and regional locations) | Whole blood donors invited to convert to plasmapheresis (declined, lapsed to WB, lapsed from panel) |
| Bagot et al. (2015) | Australia | Telephone interviews; directed content analysis | 40 | Australian Red Cross Blood Service donor panels | Whole blood donors, plasma donors, and new donors to both WB and plasma |
| Bagot et al. (2016) | Australia | Telephone interviews and focus groups; content analysis | 96 | Australian Red Cross Blood Service donor panels | Whole blood donors, plasma donors, and flexible donors with varying histories |
| Bogusz et al. (2022) | Poland | In-depth interviews; grounded theory | 10 | Regional Centre for Blood Donation and Blood Treatment (RCKiK), Lublin | COVID-19 convalescent donors with mild/asymptomatic illness who donated plasma |
| Bove et al. (2011) | Australia | Focus groups (11); grounded theory approach | 103 | Australian Red Cross Blood Service (metropolitan and regional) | Plasmapheresis donors (low-, regular-, and high-frequency) |
| Caruso et al. (2019) | Canada | Focus groups; theoretical thematic content analysis (TPB framework) | 47 | Montréal (Quebec) | Men who have sex with men (MSM), HIV-negative or unknown status, age 18+, French/English speakers |
| Charbonneau et al. (2018) | Canada | Semi-structured interviews | 50 | Héma-Québec collection centres (Montreal, Quebec City, Trois-Rivières) | Plasma and platelet donors (voluntary non-remunerated) |
| Delépine-Farvacques et al. (2017) | France | Semi-structured interviews; Theory of Planned Behaviour framework | 16 | Établissement français du sang-Bretagne (EFS Brittany) | Regular plasma donors (mean age 50, mostly men) |
| Grace et al. (2021) | Canada | In-depth interviews; inductive thematic analysis | 39 | Vancouver, Toronto, Montreal (Engage cohort) | HIV-negative gay, bisexual, queer men and other MSM |
| Holloway et al. (2022) | Canada | Semi-structured interviews | 101 | Canadian Blood Services plasma centres (Sudbury, Lethbridge, Kelowna) | Plasma donors (mostly former whole-blood donors, COVID-19 period) |
| Holloway et al. (2023) | Canada | Semi-structured interviews | 101 | New Canadian Blood Services plasma centres (Sudbury, Lethbridge, Kelowna) | Voluntary non-remunerated plasma donors (converted from whole blood) |
| Matassini et al. (2022) | Peru | Phenomenological design; telephone interviews | 11 | Hospital Nacional Edgardo Rebagliati Martins (EsSalud, Lima) | COVID-19 convalescent plasma donors (recruited via EsSalud Facebook page) |
| Ronse et al. (2018) | Guinea | In-depth interviews, focus groups, participant observation; retroductive thematic analysis | 44 | Ebola-Tx trial sites (National Blood Transfusion Centre, MSF Donka Ebola Treatment Centre, others) | Ebola survivors (donors and non-donors), CP recipients, relatives, staff |
| Sáez et al. (2020) | Guinea | Ethnographic study (participant observation, interviews, focus groups) | 45 | Plasma Mobile within Ebola-Tx trial, Conakry | Ebola survivors (CP donors), trial staff, and relatives |
| Schneider (2004) | France | Mixed methods: semi-structured interviews + quantitative survey; descriptive analysis | 310 | Établissement français du sang Nord-de-France (EFS Nord de France sites) | Plasma apheresis donors (predominantly men aged 35+, higher SES) |
| Thorpe et al. (2020a) | Australia | Semi-structured telephone interviews; thematic analysis (identity theory) | 26 | Australian Red Cross Blood Service (fixed sites) | Whole blood donors (18–65 years) recently converted to plasma |
| Thorpe et al. (2020b) | Australia | Semi-structured telephone interviews; thematic analysis | 105 | Australian Red Cross Blood Service (fixed sites) | Plasmapheresis donors (varied donation frequencies, ages 18–69) |
|  |  |  |  |  |  |
| Thorpe et al. (2021) | Australia | Narrative interviews; critical realist interpretive analysis | 17 | Australian Red Cross Lifeblood donor centres | Lapsed plasmapheresis donors (no donation ≥13 months) |
| Thorpe et al. (2022) | Australia | Focus groups, asynchronous online discussion, interviews; thematic analysis | 32 | Australian Red Cross Lifeblood anti-D programme | Anti-D plasma donors and staff (co-ordinators and medical officers) |
| Vesnaver et al. (2023) | Canada | Two-part semi-structured interviews | 27 | Canadian Blood Services donor centres (London ON; Calgary AB) | Gay, bisexual, queer men and other MSM/2SGBTQ+ eligible under revised criteria |

**Supplementary Table 2. Belief statements on plasma donation by TDF domain**

| TDF domain  (Number of distinct belief statements) | Belief statement | Frequency of each belief statement |
| --- | --- | --- |
| Behavioural Regulation (14) | I am more likely to donate when I receive clear, timely invitations or reminders. | 6 |
|  | I anchor donation in my weekly or monthly routine (e.g., “Thursday mornings are sacred”). | 3 |
|  | I feel confident in managing my donation schedule when I have control and flexibility. | 1 |
|  | I find donating easier when the process is efficient, streamlined, and provides suitable distractions. | 2 |
|  | I find it easier to donate when I have a clear schedule alternating between plasma and whole blood. | 2 |
|  | I find it easier to donate when I use scheduling tools like online appointments, mobile apps, or reminders. | 2 |
|  | I find it easier to donate when staff or systems guide me toward the product most needed at the time. | 2 |
|  | I have developed a habit or routine of donating plasma regularly. | 7 |
|  | I need extra support and reminders in my first year of plasmapheresis donation to establish a routine. | 2 |
|  | I plan my donation routine to balance whole blood and plasma donations. | 1 |
|  | I prefer to start with less frequent donations and build up gradually over time. | 1 |
|  | I stopped donating when my routine changed (e.g., job change, life demands). | 2 |
|  | Over time, I shift from being motivated by social pressure to being motivated by personal duty and solidarity. | 1 |
|  | When I can fit donation into my schedule, it is manageable. | 4 |
| Beliefs about Capabilities (3) | I am inspired to return when the process feels easy. | 1 |
|  | I believe plasma donation is as easy as or easier than whole blood once you’re used to it. | 5 |
|  | I struggle to donate because my veins are small or difficult to find. | 2 |
| Beliefs about Consequences (10) | I believe convalescent plasma donation creates hope for cures and proves survivors can contribute positively. | 2 |
|  | I believe donating plasma contributes to the health system or community. | 8 |
|  | I believe donating plasma has no negative effect on my health. | 5 |
|  | I believe donation improves how others see me (recognition, pride, belonging). | 7 |
|  | I believe plasma donation helps others and saves lives, which motivates me. | 9 |
|  | I believe plasma donation may weaken my body, health, or immunity. | 7 |
|  | I believe plasmapheresis has advantages over whole blood donation, such as donating more frequently and feeling good more often. | 2 |
|  | I believe that physical effects are minor or tolerable and do not stop me from donating. | 6 |
|  | I experience or fear physical side effects (cold sensations, strange taste, bruising, dizziness, fatigue). | 15 |
|  | I worry about contamination, infection, or permanent injury from plasma donation. | 11 |
| Emotion (10) | I feel anxious, fearful, or panicked about donating (needles, contamination, side effects). | 12 |
|  | I feel calmer and more at ease once I understand the donation process. | 6 |
|  | I feel distressed by physical sensations during donation (dizziness, nausea, tingling, fatigue). | 10 |
|  | I feel joy and satisfaction when my donation is recognized or celebrated. | 5 |
|  | I feel less anxious about donating when risks are explained and staff provide reassurance. | 5 |
|  | I feel proud, gratified, or uplifted when I donate and help others. | 7 |
|  | I feel reassured about donating when I know that physical reactions are temporary and manageable. | 4 |
|  | I feel reassured and more confident about donating when I receive clear information and transparency. | 5 |
|  |  |  |
|  | I feel unsettled or uncertain when I lack information about the process or the need for plasma. | 7 |
| Environmental Context & Resources (26) | I am more motivated when donation centers are close by or travel is convenient. | 3 |
|  | I am more willing to donate when I trust the blood collection agency or health system. | 5 |
|  | I am more willing to donate when donation centers are conveniently located and have flexible hours. | 5 |
|  | I believe for-profit plasma collection has negative consequences (cutting corners, exploitation). | 4 |
|  | I believe plasma donation is time-consuming or inconvenient compared to whole blood. | 10 |
|  | I believe plasma donation takes too long compared to whole blood. | 7 |
|  | I believe stigma or unfair rules (e.g., for gbMSM donors) harm trust and discourage donation. | 6 |
|  | I believe the process is manageable when I understand the timing and what to expect. | 4 |
|  | I believe voluntary/non-remunerated plasma donation ensures safety and fairness. | 3 |
|  | I enjoy donating in clean, comfortable, and well-resourced environments. | 3 |
|  | I feel capable of donating when the staff are skilled with needles and the process goes smoothly. | 2 |
|  | I feel encouraged to donate when staff are professional, skilled, and supportive. | 10 |
|  | I feel frustrated when donation centers are disorganized or when there are unexpected delays. | 5 |
|  | I feel hesitant when I question the motivations, fairness, or transparency of institutions. | 4 |
|  | I feel limited by time constraints, which affects my ability to commit to regular donation. | 2 |
|  | I feel more willing to donate when eligibility rules are fair, inclusive, and clearly explained. | 5 |
|  | I feel reassured when procedures are explained and equipment appears safe. | 5 |
|  | I feel restricted or excluded by eligibility rules that prevent me from donating. | 9 |
|  | I feel uncertain about my capabilities when new diseases or risks (e.g., COVID-19) are involved. | 2 |
|  | I find excessive paperwork, questioning, or bureaucracy discouraging. | 6 |
|  | I find it difficult to schedule appointments because of work, family, or unpredictable routines. | 12 |
|  | I find it discouraging when staff seem disorganized, inattentive, or unskilled. | 7 |
|  | I find it easier to donate regularly now that I am retired or have more free time. | 1 |
|  | I find it easier to donate when flexible scheduling options are available. | 4 |
|  | I find it hard to donate because donation centers are too far away or have limited opening hours. | 8 |
|  | I find long travel times to donation centers discouraging. | 5 |
| Goals (6) | I am motivated to donate plasma because I can do it more frequently than whole blood. | 2 |
|  | I feel disappointed when I cannot meet the donation goals I set for myself. | 1 |
|  | I find motivation in setting and achieving personal milestones, even when they are minor. | 2 |
|  | I see helping others as a central goal that motivates my donation. | 2 |
|  | I set personal goals for the number of donations I want to achieve. | 3 |
|  | I want to donate plasma now to make up for not donating enough when I was younger. | 1 |
| Intentions (6) | I don’t feel I need a reason to donate, I just do it when it comes up. | 1 |
|  | I feel a sense of obligation to donate because of the medical need for plasma. | 2 |
|  | I feel willing to follow instructions and donate when asked. | 2 |
|  | I intend to donate again after a positive experience. | 2 |
|  | I think donating is a good thing to do and I want to keep doing it. | 3 |
|  | I’m often too busy or lack spare time, so donation isn’t a priority. | 2 |
| Knowledge (17) | I am inspired to return when the importance of plasma is emphasized. | 1 |
|  | I don’t know enough about for-profit plasma collection and whether it is properly regulated. | 3 |
|  | I don’t know how plasma donation will affect my body or my ability to do physical activity afterward. | 2 |
|  | I don’t know how the plasmapheresis process works (e.g., needles, how blood is returned, what to expect). | 12 |
|  | I don’t know the rules about who can donate plasma, how often, or whether I can switch donation types. | 10 |
|  | I don’t know what plasma is or why it is needed. | 14 |
|  | I don’t know what the advantages and disadvantages of donating plasma are. | 4 |
|  | I feel more confident deciding to donate when I am given clear explanations and targeted information. | 2 |
|  | I find it easier to switch to plasma donation when staff explain everything clearly. | 1 |
|  | I haven’t heard much about plasma donation and don’t really know what it involves. | 6 |
|  | I need to understand how HIV risk is screened fairly across all donors before I can trust the rules. | 4 |
|  | I want more transparency about how plasma is used, where it goes, how it is sold, and what happens with profits. | 5 |
|  | When I don’t have clear information about eligibility, I’m less likely to consider donating plasma. | 3 |
|  | When I learn about plasma donation through education, events, or health studies, I am more likely to consider it. | 5 |
|  | When I learn about the uses of plasma (e.g., medicines, treatments), I am more willing to donate. | 6 |
|  | When I understand the procedure, I feel less anxious and more confident about donating. | 5 |
|  | When I understand where plasma goes and how it is used, I feel more confident about donating. | 4 |
| Memory, Attention & Decision Processes (3) | I am often too busy or short on time to keep donation top of mind. | 2 |
|  | I believe regular reminders help keep donation salient and support donation patterns. | 2 |
|  | I don’t usually remember to donate unless I am reminded by a call or letter. | 2 |
|  |  |  |
| Optimism (3) | I feel hopeful that donation programmes can challenge prejudice, acknowledge gbMSM contributions, and improve access. | 5 |
|  | I feel optimistic because my blood type or plasma (e.g., universal plasma) is especially valuable to others. | 1 |
|  | I feel optimistic because new programmes (e.g., for gbMSM) represent progress, inclusion, and reduced stigma. | 5 |
| Reinforcement (8) | I appreciate formal recognition (letters, ceremonies), though it is less important than personal gratitude. | 2 |
|  | I believe donors should receive compensation or recognition for the time and effort required. | 4 |
|  | I enjoy receiving small tangible rewards (e.g., T-shirts, pens, stickers, refreshments) that make me feel appreciated. | 6 |
|  | I feel motivated by being able to donate more frequently and have more chances to help. | 5 |
|  | I feel proud when I reach milestones (e.g., badges, awards) that recognize my donor status. | 4 |
|  | I feel reinforced when staff thank me and show gratitude for my donation. | 3 |
|  | I feel validated when I see where my donation goes and who it helps. | 3 |
|  | I worry that tangible rewards may divert resources away from patient care or the blood service. | 2 |
| Social Influences (13) | I am more willing to donate when staff recommend or encourage it. | 7 |
|  | I feel discouraged from donating when my family or close others do not support it. | 4 |
|  | I feel discouraged when I experience stigma, discrimination, or social disapproval (e.g., MSM policies). | 6 |
|  | I feel discouraged when peers or others criticize or dismiss plasma donation. | 4 |
|  | I feel discouraged when staff appear disorganized, inattentive, or undermine confidence. | 5 |
|  | I feel encouraged to donate when my family supports or participates in donation. | 3 |
|  | I feel encouraged to donate when others actively recruit or advocate for plasma donation. | 4 |
|  | I feel encouraged when I see peers, colleagues, or friends donating. | 6 |
|  | I feel encouraged when donation is framed as contributing to solidarity and collective responsibility. | 4 |
|  | I feel motivated to donate when it aligns with community norms or expectations. | 5 |
|  | I feel proud when my donation is recognized or valued by others. | 5 |
|  | I feel reinforced by building relationships with staff and other donors. | 4 |
|  | I feel unmotivated when my donation is not acknowledged or followed up on. | 3 |
| Social/Professional Role & Identity (11) | Being a donor is linked to age, gender, or social background (e.g., older men are more likely to be plasma donors). | 7 |
|  | I believe donors like me are part of a historical or social responsibility to contribute to the community or health system. | 5 |
|  | I donate because it makes me proud to be seen as helping others and contributing to society. | 6 |
|  | I donate because of my role or training in the health sector, which makes me more aware of the need for blood and plasma. | 4 |
|  | I donate because people close to me are donors or recipients, and this influences how I see my role. | 4 |
|  | I feel proud and gratified that I can contribute to society and help others by donating plasma. | 4 |
|  | I feel recognized and valued for being a donor, which strengthens my motivation. | 7 |
|  | I feel that being in a marginalized group (e.g., gbMSM) means my role as a donor is not fully recognized, and I face stigma or exclusion. | 8 |
|  | I see donation as part of my family identity or inherited values (e.g., “helping others is in my nature”). | 1 |
|  | I see my donor role as connected to trust in institutions, but I may question or resist if I feel my community’s contributions are undervalued. | 4 |
|  | I see myself as a donor, part of the donor community, and this identity motivates me to continue donating. | 9 |
