# Supplementary material for: Barriers and enablers to non‐remunerated plasma donation: A meta‐synthesis of the qualitative literature using the theoretical domains framework
Source: Vox Sang. 2026 Jul 2;121(9):1290–300. doi: 10.1111/vox.70312 (PMC13563490; doi:10.1111/vox.70312)
Supplement: Supplementary file 2 [file VOX-121-1290-s002.docx]

**Supplementary File 1. Search Strategies**

Ovid MEDLINE(R) ALL <1946 to August 23, 2023>

1	exp plasma/	31558

2	Plasmapheresis/	9214

3	plasma*.tw,kf.	1038106

4	or/1-3	1045306

5	Blood Donors/	22032

6	((blood or plasma*) adj5 (donor* or donation*)).tw,kf.	44497

7	(plasma* adj2 collection).tw,kf.	674

8	or/5-7	52744

9	4 and 8	9551

10	(enabler* or barrier* or obstacle* or hurdle* or deterrent*).tw,kf.	489429

11	motivation/ or intention/	94656

12	Health Knowledge, Attitudes, Practice/	126847

13	Altruism/	7773

14	(motiv* or intent* or altruis*).tw,kf.	345745

15	(incentive* or disincentive*).tw,kf.	37856

16	((donor* or donation*) adj3 (attitude* or belief* or conversion* or determinant* or experience* or factor* or feeling* or knowledge or opinion* or perceive* or perception* or recruit* or retent* or view*)).tw,kf.	8767

17	"surveys and questionnaires"/ or health care surveys/ or health surveys/	646328

18	(survey* or questionnaire* or interview*).tw,kf.	1687799

19	Health Promotion/	81557

20	promotion.tw,kf.	126064

21	health promot*.tw,kf.	53798

22	(promot* adj3 (strateg* or intervention*)).tw,kf.	33594

23	or/10-22	2742872

24	9 and 23	761

25	exp animals/ not humans/	5148514

26	24 not 25	747

Embase Classic+Embase <1947 to 2023 August 23>

1	exp plasma/	237247

2	plasmapheresis/	41961

3	plasma*.tw.	1391296

4	1 or 2 or 3	1429411

5	Blood Donors/	43181

6	((blood or plasma*) adj5 (donor* or donation*)).tw.	74095

7	(plasma* adj2 collection).tw.	1298

8	5 or 6 or 7	86134

9	4 and 8	17647

10	(enabler* or barrier* or obstacle* or hurdle* or deterrent*).tw.	608319

11	exp motivation/	176170

12	(motiv* or intent*).tw.	432710

13	attitude to health/	133311

14	altruism/	8684

15	altruis*.tw.	8085

16	(incentive* or disincentive*).tw.	45745

17	((donor* or donation*) adj3 (attitude* or belief* or conversion* or determinant* or experience* or factor* or feeling* or knowledge or opinion* or perceive* or perception* or recruit* or retent* or view*)).tw.	16045

18	health care survey/	24267

19	questionnaire/	903735

20	(survey* or questionnaire* or interview*).tw.	2269300

21	(recruit* adj3 strateg*).tw,kf.	6552

22	health promotion/	111499

23	promotion.tw.	142673

24	health promot*.tw.	55375

25	(promot* adj3 (strateg* or intervention*)).tw.	39407

26	or/10-25	3587204

27	9 and 26	1663

28	conference abstract.pt.	4866046

29	27 not 28	873

30	(exp animal/ or nonhuman/ or animal experiment/) not exp human/	8033105

31	29 not 30	846

APA PsycInfo <1806 to August Week 2 2023>

1	tissue donation/	2082

2	blood plasma/	6260

3	1 and 2	6

4	(plasma* adj5 (donor* or donation*)).tw.	49

5	(plasma* adj3 collection).tw.	36

6	3 or 4 or 5	85

7	exp motivation/	166881

8	intention/	20780

9	(enabler* or barrier* or obstacle* or hurdle* or deterrent*).tw.	120282

10	(motiv* or intent* or altruis*).tw.	331113

11	altruism/ or charitable behaviour/	8082

12	(incentive* or disincentive*).tw.	24292

13	(attitude* or belief* or conversion* or determinant* or experience* or factor* or feeling* or knowledge or opinion* or perceive* or perception* or recruit* or retent* or view*).tw.	2555455

14	health attitudes/ or attitudes/	42736

15	surveys/ or exp questionnaires/	39310

16	exp interviews/	19525

17	(survey* or questionnaire* or interview*).tw.	944315

18	health promotion/ or public service announcements/ or social marketing/	30103

19	promotion.tw.	49199

20	health promot*.tw.	25561

21	(promot* adj3 (strateg* or intervention*)).tw.	17617

22	(retent* or recruit*).tw.	169083

23	or/7-22	3047560

24	6 and 23	44

**Scopus – August 24, 2023**

( ( TITLE-ABS-KEY ( enabler* OR barrier* OR obstacle* OR hurdle* OR deterrent* ) ) OR ( TITLE-ABS-KEY ( motiv* OR intent* ) ) OR ( TITLE-ABS-KEY ( altruis* ) ) OR ( TITLE-ABS-KEY ( ( incentive* OR disincentive* ) ) ) OR ( TITLE-ABS-KEY ( ( survey* OR questionnaire* OR interview* ) ) ) OR ( TITLE-ABS-KEY ( promotion ) ) OR ( TITLE-ABS-KEY ( donor* OR donation* ) W/5 TITLE-ABS-KEY ( attitude* OR belief* OR conversion* OR determinant* OR experience* OR factor* OR feeling* OR knowledge OR opinion* OR perceive* OR perception* OR recruit* OR retent* OR view* ) ) ) AND ( ( TITLE-ABS-KEY ( plasma* W/2 collection ) ) OR ( TITLE-ABS-KEY ( plasma* ) W/5 TITLE-ABS-KEY ( donor* OR donation* ) ) )

801 references

**Cinahl Complete – August 24, 2023**

|  | **Query** | **Limiters/Expanders** | **Results** |
| --- | --- | --- | --- |
| S1 | (MH "Plasma+") | Expanders - Apply equivalent subjects Search modes - Boolean/Phrase | 7,518 |
| S2 | (MH "Plasmapheresis") | Expanders - Apply equivalent subjects Search modes - Boolean/Phrase | 1,136 |
| S3 | TI plasma* OR AB plasma* | Expanders - Apply equivalent subjects Search modes - Boolean/Phrase | 98,672 |
| S4 | S1 OR S2 OR S3 | Expanders - Apply equivalent subjects Search modes - Boolean/Phrase | 100,739 |
| S5 | (MH "Blood Donors") | Expanders - Apply equivalent subjects Search modes - Boolean/Phrase | 4,580 |
| S6 | TI ( ((blood or plasma*) N5 (donor* or donation*)) ) OR AB ( ((blood or plasma*) N5 (donor* or donation*)) ) | Expanders - Apply equivalent subjects Search modes - Boolean/Phrase | 6,588 |
| S7 | TI (plasma* N2 collection) OR AB (plasma* N2 collection) | Expanders - Apply equivalent subjects Search modes - Boolean/Phrase | 142 |
| S8 | S5 OR S6 OR S7 | Expanders - Apply equivalent subjects Search modes - Boolean/Phrase | 8,280 |
| S9 | S4 AND S8 | Expanders - Apply equivalent subjects Search modes - Boolean/Phrase | 1,451 |
| S10 | TI ( (enabler* or barrier* or obstacle* or hurdle* or deterrent*) ) OR AB ( (enabler* or barrier* or obstacle* or hurdle* or deterrent*) ) | Expanders - Apply equivalent subjects Search modes - Boolean/Phrase | 135,445 |
| S11 | (MH "Motivation") OR (MH "Intention") | Expanders - Apply equivalent subjects Search modes - Boolean/Phrase | 56,488 |
| S12 | (MH "Attitude to Health") OR (MH "Health Beliefs") | Expanders - Apply equivalent subjects Search modes - Boolean/Phrase | 63,735 |
| S13 | (MH "Altruism") | Expanders - Apply equivalent subjects Search modes - Boolean/Phrase | 2,748 |
| S14 | TI ( (motiv* or intent* or altruis* ) OR AB ( (motiv* or intent* or altruis* ) | Expanders - Apply equivalent subjects Search modes - Boolean/Phrase | 129,903 |
| S15 | TI ( (incentive* or disincentive*) ) OR AB ( (incentive* or disincentive*) ) | Expanders - Apply equivalent subjects Search modes - Boolean/Phrase | 16,559 |
| S16 | TI ( ((donor* or donation*) N3 (attitude* or belief* or determinant* or experience* or factor* or feeling* or knowledge or opinion* or perceive* or perception* or recruit* or retent* or view*)) ) OR AB ( ((donor* or donation*) N3 (attitude* or belief* or determinant* or experience* or factor* or feeling* or knowledge or opinion* or perceive* or perception* or recruit* or retent* or view*)) ) | Expanders - Apply equivalent subjects Search modes - Boolean/Phrase | 2,490 |
| S17 | (MH "Surveys") | Expanders - Apply equivalent subjects Search modes - Boolean/Phrase | 164,772 |
| S18 | (MH "Questionnaires") | Expanders - Apply equivalent subjects Search modes - Boolean/Phrase | 476,814 |
| S19 | (MH "Interviews") | Expanders - Apply equivalent subjects Search modes - Boolean/Phrase | 167,501 |
| S20 | TI ( (survey* or questionnaire* or interview*) ) OR AB ( (survey* or questionnaire* or interview*) ) | Expanders - Apply equivalent subjects Search modes - Boolean/Phrase | 763,036 |
| S21 | (MH "Health Promotion") | Expanders - Apply equivalent subjects Search modes - Boolean/Phrase | 77,089 |
| S22 | TI promotion OR AB promotion | Expanders - Apply equivalent subjects Search modes - Boolean/Phrase | 49,799 |
| S23 | TI health promot* OR AB health promot* | Expanders - Apply equivalent subjects Search modes - Boolean/Phrase | 29,899 |
| S24 | TI ( (promot* N3 (strateg* or intervention*)) ) OR AB ( (promot* N3 (strateg* or intervention*)). ) | Expanders - Apply equivalent subjects Search modes - Boolean/Phrase | 21,117 |
| S25 | S10 OR S11 OR S12 OR S13 OR S14 OR S15 OR S16 OR S17 OR S18 OR S19 OR S20 OR S21 OR S22 OR S23 OR S24 | Expanders - Apply equivalent subjects Search modes - Boolean/Phrase | 1,338,971 |
| S26 | S9 AND S25 | Expanders - Apply equivalent subjects Search modes - Boolean/Phrase | 194 |
