# Supplementary material for: Barriers and enablers to non‐remunerated plasma donation: A meta‐synthesis of the qualitative literature using the theoretical domains framework
Source: Vox Sang. 2026 Jul 2;121(9):1290–300. doi: 10.1111/vox.70312 (PMC13563490; doi:10.1111/vox.70312)
Supplement: Supplementary file 3 [file VOX-121-1290-s004.docx]

**Supplementary File 2. Definitions of Theoretical Domains Framework domains**

| **TDF domain** | **Definition** |
| --- | --- |
| Knowledge | Awareness of the existence of something, including procedural or factual understanding. |
| Skills | Ability or proficiency acquired through practice. |
| Social/professional role and identity | A coherent set of behaviours and personal qualities associated with one’s social or professional role. |
| Beliefs about capabilities | Beliefs about one’s ability, competence, or confidence to perform the behaviour. |
| Optimism | Confidence that desired outcomes will happen or that goals can be achieved. |
| Beliefs about consequences | Beliefs about the likely outcomes or consequences of performing the behaviour. |
| Reinforcement | Processes that increase or decrease the likelihood of behaviour through rewards, incentives, feedback, or consequences. |
| Intentions | Conscious decisions or plans to perform a behaviour. |
| Goals | Mental representations of outcomes or end states an individual wants to achieve. |
| Memory, attention, and decision processes | Processes related to remembering, attending to information, and making decisions. |
| Environmental context and resources | External circumstances, resources, systems, or environmental factors that enable or constrain behaviour. |
| Social influences | Interpersonal processes, social norms, support, pressure, or relationships that influence behaviour. |
| Emotion | Affective responses such as fear, anxiety, pride, relief, gratitude, or distress. |
| Behavioural regulation | Strategies for planning, monitoring, organizing, or regulating behaviour. |

Note: Definitions adapted from Cane et al. [20] and Atkins et al. [21].
